# Blood Glucose Levels Regulate Pancreatic β-Cell Proliferation during Experimentally-Induced and Spontaneous Autoimmune Diabetes in Mice

**DOI:** 10.1371/journal.pone.0004827

**Published:** 2009-03-16

**Authors:** Klaus Pechhold, Kerstin Koczwara, Xiaolong Zhu, Victor S. Harrison, Greg Walker, Janet Lee, David M. Harlan

**Affiliations:** Diabetes Branch, National Institute of Diabetes and Digestive and Kidney Diseases (NIDDK), National Institutes of Health (NIH), Bethesda, Maryland, United States of America; University of Bremen, Germany

## Abstract

**Background:**

Type 1 diabetes mellitus is caused by immune-mediated destruction of pancreatic β-cells leading to insulin deficiency, impaired intermediary metabolism, and elevated blood glucose concentrations. While at autoimmune diabetes onset a limited number of β-cells persist, the cells' regenerative potential and its regulation have remained largely unexplored. Using two mouse autoimmune diabetes models, this study examined the proliferation of pancreatic islet ß-cells and other endocrine and non-endocrine subsets, and the factors regulating that proliferation.

**Methodology and Principal Findings:**

We adapted multi-parameter flow cytometry techniques (including DNA-content measurements and 5′-bromo-2′-deoxyuridine [BrdU] incorporation) to study pancreatic islet single cell suspensions. These studies demonstrate that β-cell proliferation rapidly increases at diabetes onset, and that this proliferation is closely correlated with the diabetic animals' elevated blood glucose levels. For instance, we show that when normoglycemia is restored by exogenous insulin or islet transplantation, the β-cell proliferation rate returns towards low levels found in control animals, yet surges when hyperglycemia recurs. In contrast, other-than-ß endocrine islet cells did not exhibit the same glucose-dependent proliferative responses. Rather, disease-associated alterations of BrdU-incorporation rates of δ-cells (minor decrease), and non-endocrine islet cells (slight increase) were not affected by blood glucose levels, or were inversely related to glycemia control after diabetes onset (α-cells).

**Conclusion:**

We conclude that murine β-cells' ability to proliferate in response to metabolic need (i.e. rising blood glucose concentrations) is remarkably well preserved during severe, chronic β-cell autoimmunity. These data suggest that timely control of the destructive immune response after disease manifestation could allow spontaneous regeneration of sufficient β-cell mass to restore normal glucose homeostasis.

## Introduction

Type 1 Diabetes Mellitus (T1DM) is an autoimmune disease characterized by insulin deficiency and the loss of glycemia control, and is caused by the T-cell mediated destruction of pancreatic insulin-producing ß cells. It becomes clinically apparent rather late during its chronic progressive course, and has long been considered irreversible due to the assumption that β-cells have no or only limited regenerative capacity and are unable to withstand the continuing ß-cell autoimmunity. Thus, individuals diagnosed with T1DM must rely on insulin injections, a therapy of incomplete efficacy that comes with an impaired quality of life, and serious long-term side effects, that can severely limit life expectancy. Recent reports have suggested that adult β-cells might be able to expand under certain conditions [1], and experimental rodent models have confirmed this notion in general [2]. Such β-cell expansion may be due to cell duplication (mitosis) of residual β-cells [3]–[6] or neogenesis from putative intrapancreatic progenitors [7]–[9], or both; see [10] for review. In T1DM patients, the potential for β-cell proliferation has been suggested by histopathological findings [11], and persistent C-peptide production for many years after diagnosis [12]. A transient remission commonly observed shortly after T1DM onset, called the “honeymoon”, also suggests that β-cells temporarily recover functionally and/or in quantity after diabetes onset [13].

Recent studies have explored the mechanisms underlying β-cell expansion. β-cells slowly divide in adult mice and rats [14], but the process can be accelerated temporarily under physiologic [15]–[17] or pathologic conditions [3], [5], [6]. For instance, β-cell proliferation rates increase during adolescence [18], [19], pregnancy [17], [20], dietary manipulations [15], [21] that may be further stimulated in insulin-resistant states [22] and obesity, during organ regeneration after partial pancreatectomy [7], [23], or following β-cell specific cell-ablation [6]. However, no consensus has emerged regarding the proportional roles played by β-cell mitosis or neogenesis, and what regulates such β-cell expansion *in vivo* [24]. In particular, β-cell proliferative responses during autoimmune diabetes have not been studied in greater detail; in part because such efforts to increase ß-cell mass have been considered futile in the face of continued immune-mediated destruction. Other more technical factors have limited such study, i.e. the inflammatory infiltrate within the pancreatic islets and the scarcity of residual β-cells at disease onset both severely compromise the microscope-based analysis of β-cell numbers and phenotype. Thus it is unclear if residual β-cells at disease onset are responsive to signals known to stimulate β-cell expansion/regeneration under other circumstances. Such stimuli may be of local or systemic origin, and include inflammation and organ repair [25], [26], sensing a net β-cell loss [27], [28], increased glucose concentrations [29]–[32], or pathologic levels of downstream intermediates of the glucose signaling cascade [28], [33]–[35], insulin deficiency [36], [37], and a variety of incretins, secreted from sources located inside and outside of the islets [38].

Pancreas research has heavily relied on traditional techniques such as immunohistochemistry and multiparameter immunofluorescence microscopy. These methods are laborious and may suffer from a considerable heterogeneity of the disease process in the pancreas. Recognizing the limitations associated with the current assessment tools we have developed complementary flow cytometry approaches to measure small but significant changes in β-cell numbers and function during autoimmune islet destruction. Common flow cytometric techniques used to quantify (mostly hematopoetic) cell proliferation include (i) detection of increased nuclear DNA ahead of cell division, and Ab-mediated detection of either (ii) proteins whose expression is closely associated with stage-specific cell cycle progression (e.g. Ki-67 [39], phospho (p)-histone H3 [40]), or (iii) nucleoside analogs (e.g. BrdU), which are stably incorporated into newly synthesized DNA [41].

We sought to establish flow cytometry as a novel tool for pancreas research that can complement prevailing advanced microscopic techniques. Employing systematic multicolor intracytoplasmic (or -nuclear) staining strategies allowed us to examine the magnitude and regulation of islet cell growth during autoimmune diabetes. Two autoimmune diabetes models were studied, the non-obese diabetic (NOD) mouse and the rat insulin promoter (Rip) CD80×lymphocytic choriomeningitis virus glycoprotein (GP) bi-transgenic mouse, an inducible, experimental autoantigen-specific diabetes (EAD) model (see Methods). We find that the ambient glucose concentration, much more than immune cell activity or organ injury, is the primary factor associated with β-cell mitosis. Other-than-β-islet cell subpopulations do not exhibit a similar glucose dependent cell proliferation.

## Methods

### Mice

Our cytotoxic T lymphocytes (CTL)-induced EAD model is an inducible, autoantigen-specific diabetes model based on the Rip-GP×Rip-CD80 bi-transgenic mouse. In this model, the ß-cell-expressed CD80 transgene-product mediates profoundly increased diabetes susceptibility [42], and the ß-cell GP-protein provides a molecularly and immunologically well-defined target molecule suitable to sensitize antigen-specific CTL responses and diabetes development (GP, [43]). While naïve Rip-CD80+GP+ mice do not develop spontaneous diabetes, antigen-specific sensitization or transfusion of *in vitro* activated, monoclonal GP-specific, TCR transgenic CTL (p14 strain [44], Rag1-null) results in β-cell-specific autoimmune islet destruction and diabetes (termed EAD) [45]. Importantly, disease process kinetics can be controlled by the potency of GP-specific vaccinations or by controlling the transfused CTL number and activation state. The insulinoma-prone Rip-Tag2 transgenic mouse strain [46] was obtained from the repository of the Mouse Models of Human Cancer Consortium, NCI Frederick, MD, and was used as a source of tumorigenic β-cells. NOD mice (NOD/ShiLtJ) and C57BL/6J mice were purchased from Jackson Labs, Bar Harbor, ME, and either used for experiments or were further bred in house. All mice were maintained at the Division of Veterinary Resources, NIH, in accordance with the guidelines set forth by the Committee on the Care and Use of Laboratory Animals on a protocol approved by the IACUC of the National Institute of Diabetes, Digestive, and Kidney Diseases (NIDDK).

### T cell purification and in vitro activation of β-cell specific CTL

CD8+ T cells were purified from GP TCR trangenic (p14-strain, Rag1-null) by immunodepletion of I-A^b^, CD11b, and NK1.1 -expressing spleen and lymph node cells. The prepared lymphocytes were 86–95% CD8+. Purified CD8+ P14 T cells (0.5*10^6^/cm^2^) were stimulated for 3–4 days in the presence of antigen-presenting cells (APC) preloaded with its agonist peptide (GP aa 33–41 at 0.1 µM for 1 h,) at a T/APC ratio of 20. These APC were C57BL/6-derived pancreatic fibroblast cell lines, that had been cultured in DMEM 10% FCS supplemented with 2 ng/ml recombinant murine IFNγ to upregulate MHC class I expression for the final 1–2 days prior to T cell co-culture. 48 h after T cell activation, cultures were supplemented with IL-2 (2 ng/ml). On day 3 of culture virtually all viable cells had an activated phenotype and by flow cytometry were >98% CD8 and TCR-transgene positive (data not shown).

### Diabetes induction and diagnosis

We initiated ß-cell-specific islet destruction in Rip-CD80×Rip-GP bi-transgenic mice at a median age of 10.3 weeks by adoptively transferring (i.v.) CTL (10^6^/mouse) as described above. Fibroblast-stimulated d3CTL typically mediate a slowly progressive ß-cell destruction in the (CTL-induced) EAD model (diabetes onset after 53+/−9 d). NOD mice were allowed to spontaneously develop diabetes (diabetes onset for both diabetes models is shown in **Supporting Information Figure S1**). Prior to the expected time of diabetes onset, non-fasted mice were checked daily for urine glucose, and diabetes was confirmed by blood glucose (BG) readings above 13.3 mM (240 mg/dl) on two consecutive days. The term pre-diabetes has been used rather broadly, randomly encompassing non-diabetic mice with no attempt to predict the stage of diabetes development more precisely (e.g. using histopathology or glucose tolerance testing).

### Induction of diabetes control

We used three different ways to temporarily or permanently restore normoglycemia with or without control of autoimmunity:

1. **CD8 T lymphocyte immunodepletion:** CD8 T lymphocyte-depleting mAb (anti-CD8, clone YTS169, [47]) was injected i.p. twice 48 h apart (500 and 200 µg/mouse, respectively). This treatment led to gradual diabetes remission and normoglycemia (urine glucose-negative and BG<∼11.1 mM [200 mg/dl]), which lasted for at least 7 consecutive days in 6 of 9 mice. Prior experience has shown that remissions are not permanent, with most mice developing recurrent hyperglycemia within 1–2 weeks, i.e. when the depleted T cells recover.
2. **Insulin pellets:** Exogenous insulin was administered via insulin-releasing pellets (about 1 mm×4 mm in size releasing approx 0.1 U/24 h, LinShin Canada Inc., Toronto, Canada). Under short-term anesthesia, the left flank skin was punctured using a 14G needle, and a pellet applicator was used to place usually a single insulin pellet s.c. Normal BG levels were typically restored within 12 h, and generally “normal” BG concentrations were observed, typically for more than a week. In some cases, insulin pellets were surgically removed after 3 days of normoglycemia, which instantly led to recurrent hyperglycemia.
3. **Islet transplantation:** C57BL/6 islets were used to restore normoglycemia in congeneic Rip-CD80+GP+ mice. We have previously established that these wild type islets do not constitute a target for autoreactive T cells, since they lack both the costimulatory CD80 molecule and the GP autoantigen. Such islet grafts permanently cure diabetic Rip-CD80+GP+ mice with no histological evidence of congeneic graft rejection. Islets were transplanted under the left kidney capsule using a retroperitoneal approach. Briefly, under isoflurane anesthesia, the left kidney was exposed, and a pouch was formed between the capsule and the kidney parenchyma of about 7×12 mm size. 300–400 islets (cultured overnight in standard culture medium: DMEM 4.5 g/L Glucose, 10% FCS) were transferred into a sterile silicon laboratory tubing (30 cm length, 0.51 mm diameter, Silastic, Dow Corning, Midland, MI). By a brief spin, islets were collected at one end of the tubing, which was then slipped into the kidney pouch, and the islets were deposited. The pouch was sealed using tissue glue (Vetbond, 3M, St. Paul, MN). The wound was closed, and pain relief was provided for 2 days (banamine 1.5 ug/g BW twice daily). BG levels dropped to normal levels within several hours postoperatively. Mice remained normoglycemic for the entire experiment; that is the mice had no glucosuria and random BG values were essentially normal (7–8 mM, see **Supporting Information Table S1**).

### Islet isolation

Pancreatic islets were isolated by standard techniques, with minor adaptations. Briefly, pancreata from naïve mice or from mice at various stages of autoimmune diabetes development, were inflated via bile duct cannulation and retrograde pancreatic duct injection of 3–4 ml of ice-cold collagenase type XI (1 mg/ml in HBSS, Sigma, St. Louis, MO). Following digestion (37°C, 14 min), pancreata were broken apart by aspirating through a 14G needle, then filtered through a metal strainer (0.8 mm). Pancreas suspensions were then subjected to buoyant density gradient centrifugation (14–15% Optiprep, Accurate Chemicals, Westbury, NY), followed by handpicking of all discernible islets. Upon removal of islets, the remaining viable pancreatic cells were collected and termed the islet-depleted fraction, containing sub-islet-sized endocrine clusters and single endocrine cells.

### Islet dissociation and hormone staining

Isolated islets were dissociated into single cell suspensions after washing in 2 mM EDTA/PBS, then incubating for 10 min at ambient temperature in Ca^2+^ free PBS supplemented with 0.025% trypsin, and finally by careful pipeting. Dissociated islet cells prepared from prediabetic or diabetic mice were stained for CD45, to identify infiltrating lymphocytes, followed by washing, and immediate single step fixation and permeabilization (4% PFA, Electron Microscopy Sciences, Hatfield, PA, 0.1% saponin, Fluka Chemicals, Buchs, Switzerland, in PBS, 30 min room temperature). After removing PFA by washing in 0.1 saponin/1% BSA/PBS, islet cells were stained intracytoplasmically for 25 min with Ab to insulin (1∶300, guinea pig, DAKO, Carpinteria, CA). For multiple hormone stainings, glucagon and somatostatin mouse IgG1 mAb (respectively K79bB10, Sigma, and SOM018, Ab core facility, Beta Cell Biology Consortium [BCBC], Denmark) were used in conjunction with Zenon (pre)labeling technology for mouse IgG1 (AlexaFluor488 and Pacific Blue conjugates, respectively, Invitrogen, Carlsbad, CA). Highly cross-absorbed, second-step polyclonal Ab, anti-guinea pig-Cy5 was from Jackson ImmunoResearch (West Grove, PA). After the final wash in 1%BSA/saponin, cells were post-fixed in 1% PFA and acquired using a CyAn ADP flowcytometer and Summit V 4.3 Software (Beckman-Coulter, Miami, FL). Electronic gating was set to include viable cells on the basis of forward scatter vs. side scatter while doublet-exclusion gating was applied to eliminate non-dissociated islet cell couplets on the basis of pulse width patterns for each islet cell subset (color). The numbers of β-cells of all acquired and analyzed samples (2–3 samples per mouse islet preparation) ranged from 4,021–37,213 for naïve, and from 754–18,950 for diabetic mouse islets.

### Quantitative DNA staining

Quantitative DNA staining (5 uM, Vybrant DyeCycle Violet, Invitrogen) was used to enumerate cells with increased DNA content, indicative of cell cycle S- or G_2_/M phase. Vybrant DyeCycle violet staining reagent was chosen for its quantitative DNA staining properties (DNA-binding induces a linear increase in fluorescence), and spectral characteristics using a violet laser (excitation/emission were 405 nm and 450±20 nm, respectively) to avoid overlap with other fluorescent dyes excited by different lasers in multicolor applications. To ensure single cell analysis, a doublet-exclusion strategy was applied involving linear signal area versus signal peak (height) gating in the presence of a quantitative DNA stain and the flow cytometer's hydrodynamic sample stream focusing provisions. The algorithm underlying electronic doublet exclusion strategies is well established [48], taking advantage of couplet orientation during hydrodynamic focusing (i.e. when sheath fluid forces the sample stream through the nozzle to the laser intersection point). While doublet exclusion gating eliminated nearly all double hormone positive cells (a ∼20 fold reduction), it did not significantly alter the relative endocrine cell frequencies and the calculated cell ratios (**Supporting Information Figure S2**). As nuclear staining intensity doubles when proliferating cells transition through the phases of the cell cycle, the G_0_/G_1_ subset's coefficient of variation (CV, representing the normalized measure of dispersion of single event signals) becomes critically important to distinguish the G_0_/G_1_ to S-Phase transition. Typically, cytoplasm-stripped, isolated nuclei are used for cell cycle analysis, since purified nuclei exhibit a smaller CV. However, identifying ß-cells in a heterogeneous islet cell suspension requires intracytoplasmic insulin staining, resulting in a greater CV due to the increased autofluorescence and cytoplasmic fluorescence background. Therefore, to minimize distorting early S-Phase cell counting, we arbitrarily defined the linear fluorescence intensity of nuclear DNA in proliferating β-cells as ≧1.4-fold of the G_0_/G_1_ mean linear fluorescence intensity. Thus, the 1.4-fold mean linear G_0_/G_1_ fluorescence cutoff while arbitrary, estimates the number of cells progressing through S-Phase until mitosis completion.

### BrdU-labeling and detection by flow cytometry

BrdU (Fluka Chemicals) was either supplied continuously for 7 days in drinking water (1 mg/ml), or injected i.p. The latter involved either 1 injection (1.5 mg/mouse), or daily injections on three consecutive days prior to islet isolation (first injection 1.5 mg, and 1 mg each for the following 2 days). BrdU staining for flow cytometry of fixed dissociated islet cells required pre-treatment with DNase I (0.1 mg/ml [approx. 57 Kunitz Units/ml], Sigma, in HBSS/0.1% saponin (30 min at 37°C), followed by staining for 25 min at 4°C with anti-BrdU mAb (clone PRB-1 conjugated to Alexa Fluor 647, Invitrogen).

### Statistical analysis

An independent Student's T Test (one-tailed) was chosen to test the significance of increases or decreases between data sets (endocrine cell ratios), and the linear regression analysis was described using the Pearson product moment correlation coefficient, r.

## Results

### Flow cytometry demonstrates that autoimmune diabetes stimulates ß-cell cycle progression

**Figure 1:**
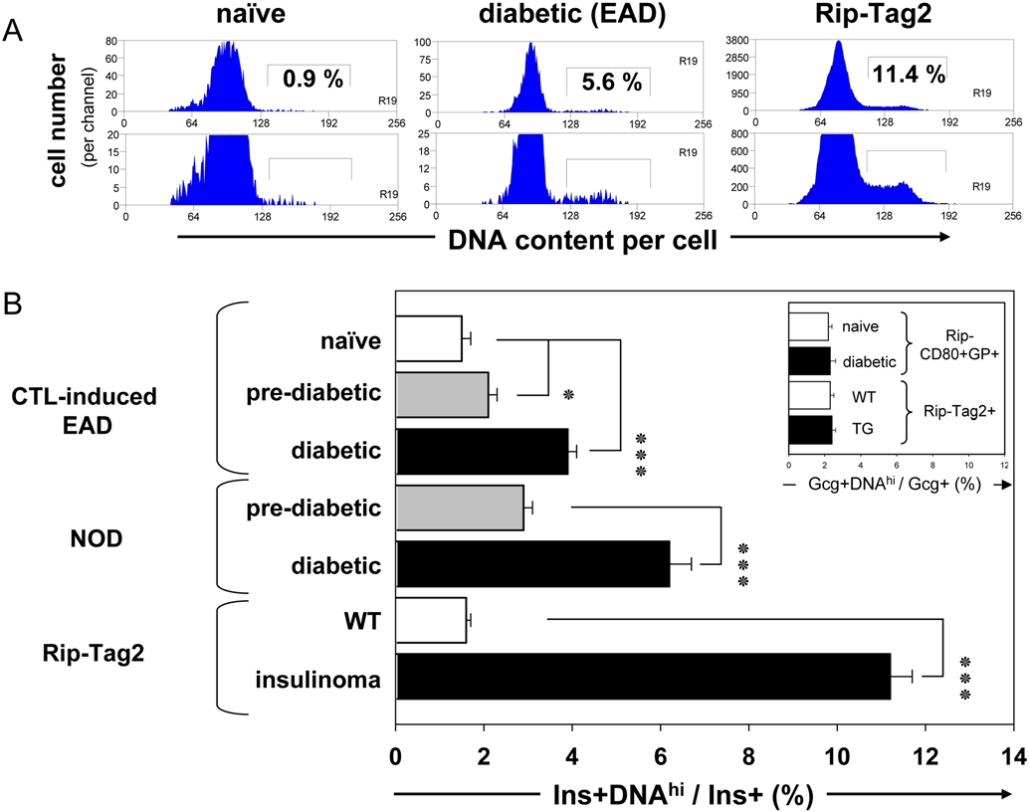
Assessment of β-cell replication by quantitative staining of nuclear DNA and multicolor flow cytometry. Purified islets from naïve and diabetic EAD and NOD mice, and insulinoma-developing Rip-Tag2 Tg mice were dissociated into a single cell suspension, then were fixed and stained for insulin (Ins) and glucagon (Gcg). Nuclei were stained using a quantitative nuclear dye (Vybrant DyeCycle violet). A: Nuclear DNA staining profiles of gated Ins+ β-cells. The range of different frequencies of DNA^hi^ β-cells from groups of mice is shown. These were non-diabetic, naïve (range 0.8–1.9), diabetic EAD (range 2.5–5.7), and Rip-Tag2 transgenic mice (range 7.4–14.6). The lower panel represents a magnified y-axis range to better illustrate the DNA^hi^ β-cells. B: Statistical analysis of increased β-cell DNA content displayed as percentage of overall Ins+ cells (mean±SE): EAD (naïve, 1.5±0.2, n = 15; and CTL-induced pre-diabetic, 2.1±0.2, n = 12; and diabetic, 3.9±0.3, n = 20); NOD (pre-diabetic, 2.9±0.2, n = 13; diabetic, 6.2±0.5, n = 6), and Rip-Tag2 (WT, 1.6±0.1, n = 25; transgenic, 11.2±0.5, n = 16). Significance levels: (*) p = 0.026, and (***) p<0.0001. The inset depicts a similar DNA content analysis on Gcg+ α-cells of EAD and Rip-Tag2 mice, showing no detectable difference in α-cell DNA content among mice.

Reasoning that adapting flow cytometry-based techniques to pancreas research would greatly facilitate a more quantitative analysis of β-cell proliferation in islets undergoing autoimmune destruction, we have developed protocols to isolate islets from naïve and diabetic mice, dissociate them into single cells, and identify their endocrine cell lineage by co-staining for intracytoplasmic insulin, glucagon, and somatostatin (data not shown). **Supporting Information Figure S3** illustrates electronic gating strategies to identify islet β-cells, based sequentially on CD45+ hematopoetic-lineage cell exclusion, insulin-staining, and eliminating not fully dissociated islet cell clusters using the hydrodynamic focusing-based, electronic doublet exclusion gating strategy on nuclear fluorescence. As illustrated in **Figure 1A**, we determined the fraction of cells with increased DNA content (ranging from less than 1% to more than 10%) using a quantitative DNA-intercalating dye, and electronically-gating on β-cells from healthy, naive (left), and recently diabetic mice (middle), and among Rip-Tag2 transgenic animals that develop insulinomas (right) [46]. Quantitative analysis (**Figure 1B**) demonstrates that islet cells isolated from newly diabetic EAD mice had a higher proportion of β-cells with increased DNA content (3.9%, range 2.5–5.7, n = 20) compared to naïve mice (1.6%, range 0.8–2.4%, n = 25), suggesting that diabetic mice have a greater proportion of β-cells with ongoing DNA synthesis and cell cycle progression. Expectedly, tumor-prone Rip-Tag2 transgenic mice, when tested well before they developed clinical symptoms of (transgene-mediated) insulinomas, regularly showed a substantially increased proportion of β-cells with the increased DNA content (11.2%, range 7.4–14.5, n = 16). Evidence supporting increased β-cell cycle progression at diabetes onset was not unique to our CTL-induced EAD model; we found similar results using islets isolated from spontaneously diabetic NOD mice (6.2%, range 4.0–7.3, **Figure 1B**). Pre- or non-diabetic NOD mice of a comparable age but with incompletely characterized glucose tolerance had a somewhat increased proportion of β-cells with elevated DNA content (2.9%, range 1.8–4.4). Importantly, in rodent models of both autoimmunity and ß cell tumor development, we found increased DNA content only in β-cells and not in α-cells (**Figure 1B inset**), based on glucagon staining and a comparable gating strategy. While the DNA content analysis for determining proliferation enjoys the important advantage of not having to label tissue *in vivo*, the technique does suffer from technical and/or biological limitations. These potentially confounding factors include that: (1) DNA is partially degraded in the early stages of islet cell apoptosis, which may occur during islet inflammation or the islet isolation process, (2) the S-Phase cell DNA quantification requires establishing a somewhat arbitrary cutoff, (3) dysfunctional islet cells may be arrested in the G_2_/M phase after completing S-phase but before they undergo cell division, and (4) doublet exclusion efficacy may vary. Cellular uptake of the nucleoside analog BrdU during S-Phase transition would, unlike DNA content, likely be more stringent in labeling proliferating cells, but the technique does not label G_2_/M-Phase cells, and has further limitations. For instance, if BrdU is given some time before islet isolation, the amount incorporated into DNA reflects the cumulative history of cell cycles completed during the BrdU exposure. Consequently, BrdU-labeling experiments (especially those in which the BrdU was dosed some time before euthanasia) cannot distinguish between endocrine cell replication and progenitor cell replication followed by later differentiation into an endocrine phenotype. Hence, we have used the term “proliferation” or “replication” more broadly to accommodate both possibilities.

### BrdU+ ß-cell detection by flow cytometry and ß-cell DNA content analysis yield comparable results

**Figure 2:**
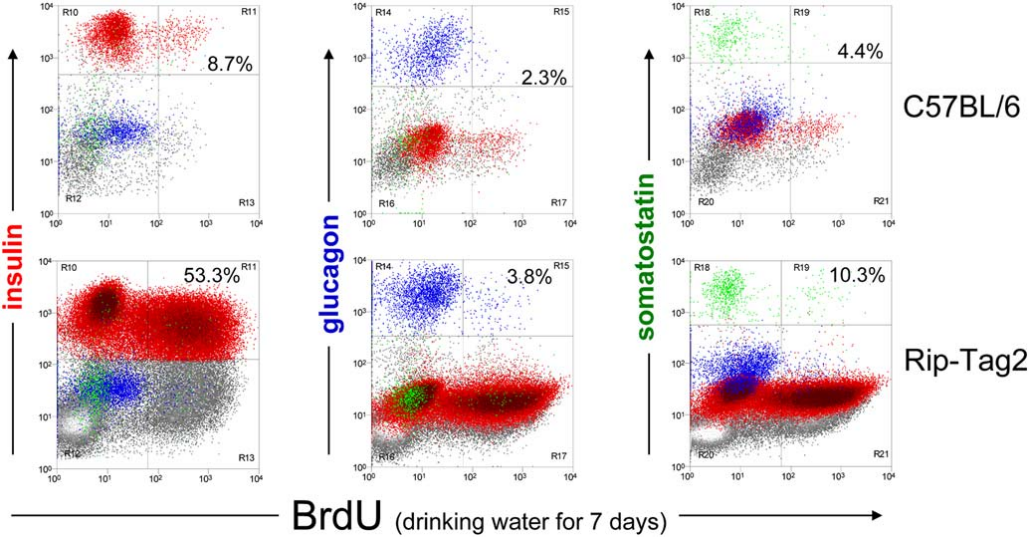
Detection specificity of multiparameter flow cytometry of continuously *in vivo* BrdU-labeled islet cells. Mice aged 7–9 weeks were given BrdU at 1 mg/ml in their drinking water continuously for 7 days. Islets were isolated, dissociated, and 4-color stained for insulin (red), glucagon (blue), somatostatin (green), and for incorporated BrdU. BrdU incorporation (x-axis) was plotted for the endocrine islet cell subsets from naïve, C57BL/6 (top panel), and insulinoma-developing yet non-symptomatic Rip-Tag2 Tg mice. Percentages indicate the relative frequency of BrdU-stained, single cells among the particular endocrine subset identified by hormone staining. Note that depending of the stage of insulinoma development, the frequency of glucagon+ α-cells and somatostatin+ δ cells are correspondingly reduced among all islet cells acquired from purified islets of insulinoma carrying mice.

In accordance with numerous studies using flow cytometry to detect BrdU-labeled hematopoetic cells, **Figure 2** demonstrates the feasibility of this approach using dissociated islet cells (see **Supporting Information Figure S2** illustrating the gating strategy applied to islet cell BrdU staining). Analysis of 8–10 week-old WT mice given continuous BrdU for 7 days in their drinking water labeled just 8.7% of their β-cells (**Figure 2**). In contrast, age-matched Rip-Tag2 transgenic mice incorporated BrdU in more than half their β-cells during the same labeling period. The BrdU uptake in insulinoma-prone mice was β-cell specific; i.e. both WT and insulinoma-prone mouse islet α- and δ-cells incorporated very little BrdU. However, we found that endothelial cells, and perhaps other islet cell subsets, markedly expanded during insulinoma formation (data not shown), which accounts for the increased BrdU incorporation present in non-endocrine islet cells (**Figure 2**, lower panel, lower right quadrants). We also noted the 2-fold increase in proliferating somatostatin-positive δ cells in the Rip-Tag2 transgenic mice, but we did not further explore the significance of this finding.

**Figure 3:**
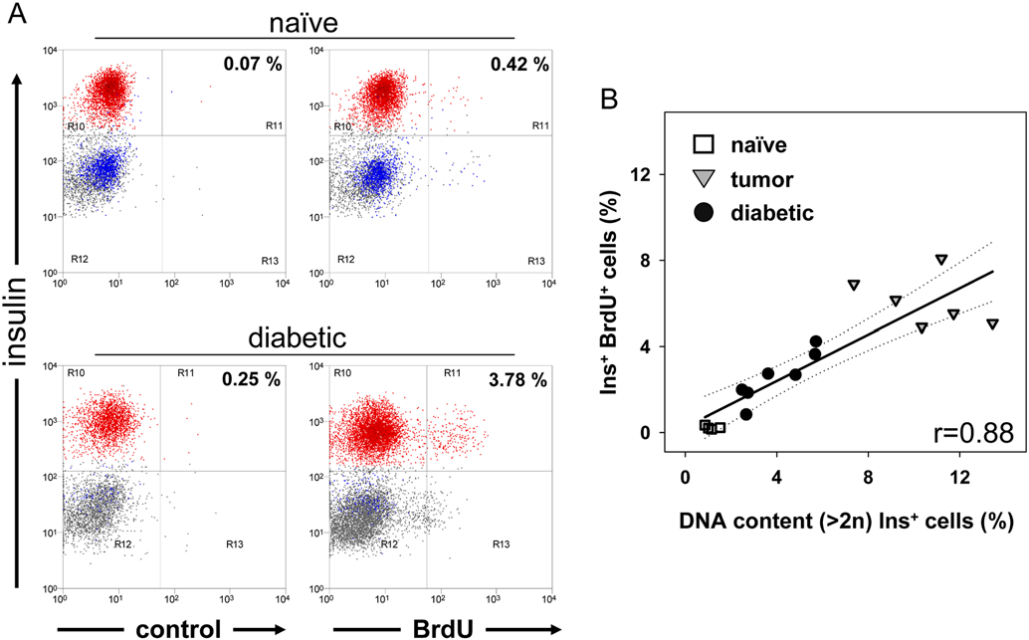
Single-pulse BrdU-labeled β-cells are more frequent in diabetic mice, and correlate with ß-cell DNA-content measurements. Naïve and diabetic mice around 20 weeks of age received a BrdU injection (10 mg/ml at 2.5 mg/mouse, i.p.). Islets were isolated the following day and 5-color stained (3-hormones, CD45+ leucocytes, and incorporated BrdU). A: Representative example of a naïve (top panel), and a newly diabetic CTL-induced EAD mouse (bottom panel). BrdU-staining is shown on the right panel, while isotype/fluorochrome controls are shown on the left. Note that BrdU incorporation (right panels) upon short-term exposure in naïve islets β-cells is very low, but increases in diabetic mice. B: Correlation plot between BrdU pulse-labeling and the frequency of increased DNA content among islet β-cells. Each symbol represents islets of a single mouse, aliquoted for independent staining of BrdU and DNA content. Three different groups of mice were tested: Rip-CD80+GP+ (naïve, open square; and diabetic, closed circles), and insulinoma-developing Rip-Tag2 mice (open triangles). Correlation coefficient r = 0.88; 95% confidence interval (dashed lines).

For the study of diabetic mice, BrdU via drinking water is inappropriate because of the expected increased BrdU-uptake due to the diabetes-associated polydipsia. We therefore injected the BrdU i.p. to pulse-label islet cells, and as expected found a substantially lower percentage of labeled endocrine cells (0.42%, **Figure 3A**) compared to the 7d-drinking-water continuous labeling technique (8.7%). We can suggest at least three explanations for labeling fewer cells using the pulse-labeling technique. One, BrdU labeling during DNA synthesis is cumulative because the label is retained by daughter cells even when they return to quiescence after cell division. Two, BrdU availability differs when the agent is given in drinking water or by i.p. injection; after a bolus injection, BrdU half-life is less than two hours [49]. Three, somewhat older WT mice (∼20 wks) were used in the pulse-labeling experiment to appropriately match the generally older EAD mice at diabetes onset (**Figure 3A**, see also **Methods** and **Supporting Information Figure S3**), and advanced mouse age has previously been shown to correlate with decreased β-cell BrdU uptake [14]. Thus, the BrdU studies using diabetic mice support the conclusions drawn using DNA content determination; i.e. compared to mice with normoglycemia, diabetic mice have a substantially higher frequency of BrdU+ β-cells (9-fold). To directly compare DNA content and BrdU labeling assays for ß cell mitotic activity, we studied individual mouse islet cells in a split sample approach. As shown in **Figure 3B**, we observed a tight correlation whether we studied naïve mice, or those with diabetes or incipient insulinomas (r = 0.88). To further correlate increased DNA-content with other markers of cell cycle progression we stained for phospho (p)-histone H3 [40] and Ki-67 [39]. **Supporting Information Figure S4** demonstrates the feasibility of β-cell staining for Ki-67. The cell cycle associated protein Ki-67, whose function is still poorly understood, is generally expressed during the active cell cycle (G_1_ through M-Phase) [39]. Ki-67 detection correlated incompletely with increased DNA-content, as has been observed in islet-infiltrating lymphocytes, and in a β-cell tumor line (Min6, data not shown). The incomplete correlation may result from previously discussed limitations of the DNA-content assay and/or from Ki-67's variable expression density during the cell cycle. We also attempted to stain β-cells directly *ex vivo* for the M-Phase-specific marker p-histone H3, typically present in a small minority of proliferating cells, but if such cells exist, our system was insufficiently sensitive to detect them (**Supporting Information Figure S4**).

### Diabetic mouse β-cells undergo regulated proliferation in situ, and maintain insulin expression during replication

We compared flow cytometry based BrdU+ β-cell frequencies with conventional histological techniques used to enumerate BrdU+ β-cells. **Table 1** illustrates that BrdU+ β-cell numbers found in either naïve or diabetic mice were quantitatively comparable using both techniques, further validating our novel flow cytometric approach. We sought to rule out the possibility that BrdU incorporation might be explained by stress-induced DNA damage/repair or cellular dysfunction in the highly inflammatory environment at diabetes onset. We found that single dose BrdU-labeled diabetic mouse ß cells display a homogenous, bright, and nuclear BrdU immunofluorescence, strongly suggesting that BrdU incorporation indeed happened during S-phase (**Supporting Information Figure S5**, and **Supporting Information – Methods S1**). Interestingly, we observed several colocalized BrdU-positive β-cell pairs or small clusters by confocal microscopy suggesting that these cells did undergo cell division *in situ* within the 3 day BrdU pulse-labeling period **(Supporting Information Figure S6)**. Further, we tested if β-cells incorporate BrdU during S-Phase but did not complete cell division, a process known as endoreduplication [50]. Endoreduplication results in cells maintaining enlarged nuclei that contain (BrdU-labeled) DNA in the amount of 4N or greater. A closer examination by immunofluorescence microscopy of pancreatic sections from diabetic mice however, did not reveal differences in the average β-cell nuclear cross-sectional area when BrdU+ nuclei were compared to neighboring BrdU- β-cells (**Supporting Information Figure S7**), thus arguing against a prominent role for β-cell endoreduplication during autoimmunity. Using flow cytometry we also determined if β-cells down-regulated their insulin content during cell cycle progression. Comparing β-cell mean insulin fluorescence intensity (an estimate of cytoplasmic insulin content) in cells separated for low DNA-content (DNA^lo^ or ≈2N) and DNA^hi^ (>2N), we found comparable insulin staining intensity in diabetic EAD (DNA^hi^∶DNA^lo^ = 1.09, n = 15), diabetic NOD mice (1.02, n = 5), and tumor-developing Rip-Tag2 mice (1.08, n = 18). Thus, β-cells appear to maintain their insulin content during replication.

**Table 1:** Blood glucose selectively controls ß-cell proliferation in islets of diabetic mice.

	naïve				diabetic			
	BrdU	-label: 1 d a	BrdU	-label: 3 d	BrdU	-label: 1 d	BrdU	-label: 3 d
	(n)	BrdU+ (%)^b^	(n)	BrdU+ (%)	(n)	BrdU+ (%)	(n)	BrdU+ (%)
**IF** c	2	0.4	2	0.5	2	1.8	4	5.5
**FCM**	4	0.2	7	0.1	8^d^	2.3	14^d^	5.7

a BrdU-labeling by injection (i.p.), either 1 day (1 d), or on 3 consecutive days (3 d) before pancreas harvest.

b percentage of BrdU+ cells among insulin+ cells.

c immunofluorescence microscopy scoring of ≧ 47 individual islets or small endocrine clusters of at least 2 spatially separated pancreas sections per mouse. Total β-cells scored: (naïve mice: 1,830; diabetic mice: 944)

d mice analyzed include islets in Figure 3, and Table 2, respectively.

### β-cell proliferation at autoimmune diabetes onset is regulated by BG concentrations

The methodology to measure islet cell proliferative activity allowed us to uncover potential forces underlying its regulation at diabetes onset. We sought to distinguish metabolic cues stimulating ß cell replication (most obviously severe hyperglycemia) from local factor(s) present during organ injury and/or chronic inflammation. To differentiate these mechanisms, we restored normoglycemia in CTL-induced EAD diabetic mice in various ways designed to supply insulin by either the regenerating endogenous ß-cells (afforded by deleting the ß-cell specific CTLs), or entirely from extrapancreatic sources (continuously releasing insulin-pellets, or congeneic islet grafts). Because the primary, CTL-recognized autoantigen in the EAD model is the β-cell transgene-expressed GP protein, we assumed that neither continuous exogenous insulin, nor congeneic (but non-transgenic) islet grafts would significantly alter the inflammatory environment or the anti-GP autoimmune response in the pancreas during the short 4–7 day course of the experiments.

**Figure 4:**
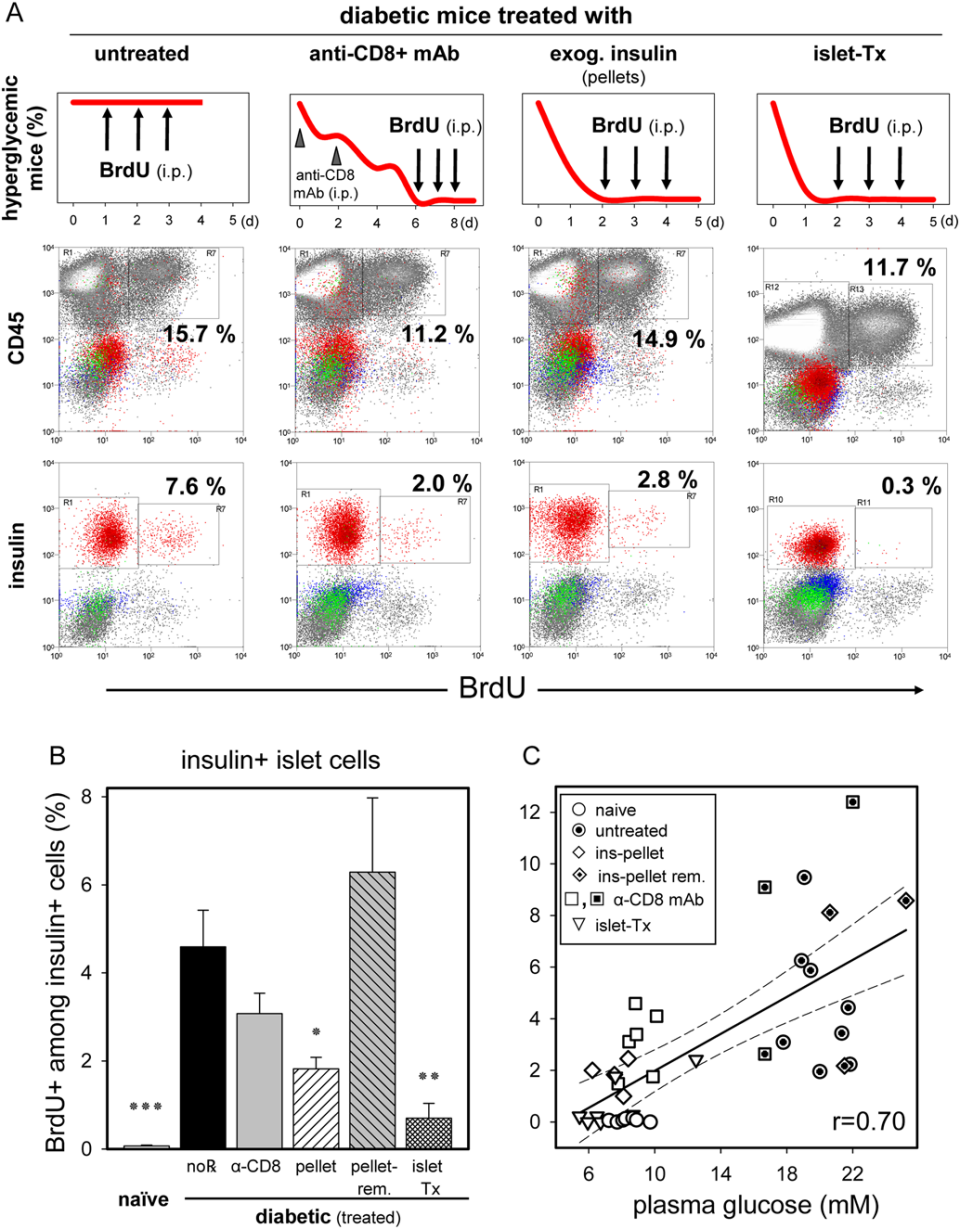
Reduced islet β-cell BrdU uptake in diabetic mice treated to restore normoglycemia. Diabetic Rip-CD80+GP+ mice were randomly assigned for treatment groups to normalize BG levels, followed by BrdU pulse labeling (3 doses indicated by arrows of 1.5, 1.0, 1.0 mg/mouse i.p. over the final 3 days of the experiment), and islet cell analysis by flow cytometry. A: Treatment regimen, expected and exemplified results are illustrated in columns. β-cell BrdU incorporation was increased in the presence of untreated hyperglycemia (lower panel). Note that both the abundance and BrdU incorporation of islet infiltrating CD45+ leucocytes was essentially unchanged among treatment groups (middle panel). B: BrdU-uptake of islet β-cells is controlled by BG levels. Groups of treated diabetic mice (no℞, n = 8; anti-CD8 mAb, n = 6; s.c. insulin pellet, n = 4; ins-pellet removed, n = 3; islet-Tx, n = 7) were compared to age-matched, naïve mice (n = 7). Results are shown as mean±SE, with the following significance levels: (*) p<0.05, (**) p<0.01, and (***) p<0.001. C: Correlation plot between the final 3-day average random BG readings (x-axis) and the frequency of BrdU+ islet β-cells (y-axis). Each symbol represents a single mouse, and all mice, regardless of treatment group are included. Correlation coefficient r = 0.70; 95% confidence interval (dashed lines).

**Figure 4A** illustrates how hyperglycemia resolved as a result of the different treatments (upper panel, **Supporting Information Table S1** provides the treatment group's average BG concentrations recorded during the 3-day BrdU labeling period), and exemplifies the concomitant detection of BrdU uptake by infiltrating leucocytes (middle panel) or non-lymphocytic islet cell subsets (lower panel). **Figure 4B** quantifies the various treatments' impact on endogenous β-cell proliferation. Exogenous insulin (s.c. pellets or islet-transplantation) restored normoglycemia in every mouse, whereas depleting CD8+ T cells transiently halted direct CTL-mediated β-cell killing so gradually restored normoglycemia in the majority of diabetic mice (responders), with remissions typically lasting for more than one week. Regardless the treatment (T cell depletion by anti-CD8 mAb, exogenous insulin by s.c. insulin pellets, or islet transplantation), restoring normoglycemia to diabetic mice profoundly blunted the increased β-cell proliferation observed in newly diabetic, hyperglycemic mice. In fact, using our CTL-induced EAD model, we found a robust correlation (r = 0.70) between β-cell proliferation (BrdU pulse-labeled for 3 days prior to islet isolation) and the average random BG level during the same period prior to euthanasia (**Figure 4C**). The importance played by the BG level was further emphasized in mice that were not restored to normal BG levels (non-responders) by the anti-CD8 antibody treatment; in such mice with persistent hyperglycemia, β-cell proliferation remained elevated (8.0±2.3%, n = 3, data not shown). This also argues against the anti-CD8 antibody causing an inhibitory or otherwise toxic effect on β-cell proliferation. Most importantly, residual β-cells remained responsive to changes in BG levels. For instance, if we restored normoglycemia using insulin pellets and thereby decreased β-cell proliferation, removing the pellets rapidly resulted in both hyperglycemia and increased β-cell proliferation (**Figure 4B**, “pellet-rem”).

**Figure 5:**
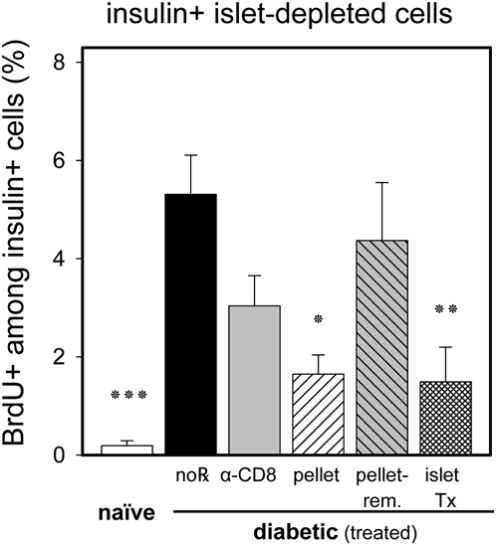
BrdU incorporation of β-cells in small pancreatic endocrine cell clusters also corresponds to BG concentrations. Sub-islet sized clusters, often thought to be recently differentiated and/or proliferated endocrine cells were examined for BrdU uptake. The same treatment groups as in Figure 4B were analyzed and displayed accordingly. Results are shown as mean±SE, with the following significance levels: (*) p<0.05, (**) p<0.01, and (***) p<0.001.

Our data suggest that hyperglycemia, rather than local inflammatory processes, was primarily responsible for increased β-cell proliferation at diabetes onset. We further tested this hypothesis by comparing the mitotic response of β-cells within islets and those found in extra-islet endocrine tissue (termed sub-islet sized endocrine clusters). These endocrine cells may be more immediately targeted and therefore more vigorously attacked by β-cell-specific CTL, an assumption based on histological findings. That is, we invariably find inflammatory cells in these small clusters while at diabetes onset, CTLs have invaded large individual islets to a variable extent. Indeed, consistent with the hypothesis that BG levels regulate β-cell replication in autoimmune diabetes, and using the same mice shown in **Figure 4B**, we observed virtually identical proliferative responses to therapeutic normoglycemia in residual β-cells found in sub-islet-sized endocrine clusters, compared to those found in large islets (**Figure 5**). While we believe these small endocrine clusters comprise an anatomical compartment separate from traditionally recognized large islets, they could also represent over digested or fragmented large islets.

### Spontaneously diabetic NOD mouse β-cell proliferation was similarly controlled by BG concentrations

**Figure 6:**
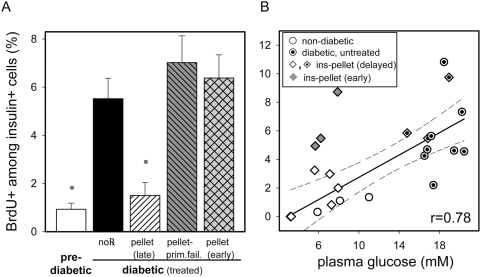
Glycemia regulates β-cell proliferation in spontaneously autoimmune diabetic NOD mice. A: BrdU uptake by residual β-cells of NOD mice is controlled by BG levels. Newly diabetic NOD mice were randomly treated with: no℞, n = 8; exogenous insulin (pellet) and BrdU labeled day1–3 (early), n = 3, crosshatched grey bar, or BrdU labeled day 4–7 (late), n = 6, hatched; insulin pellet treated mice that did not restore normoglycemia (prim. fail.), n = 4, hatched grey bar, and were compared to pre-diabetic NOD mice, n = 3, open bar. Results are shown as mean±SE, with significance levels: (*) prediabetic: p = 0.016, and insulin pellet (late): p = 0.0051, respectively. B: Correlation plot as in Figure 4C. Each symbol represents a single mouse, and all mice, regardless of treatment group are included. Correlation coefficient r = 0.78; 95% confidence interval (dashed lines). Note, that NOD mice that restored normoglycemia upon insulin pellet, and were immediately BrdU labeled (pellet early) maintain increased β-cell proliferation. These mice are shown in the plot (⧫) but were omitted from correlation coefficient calculation.

We also measured the β-cell proliferation rate in the commonly studied NOD mouse, a spontaneous autoimmune diabetes model. As shown in **Figure 6**, β-cells from newly diabetic NOD mice exhibited increased proliferation (5.5±0.9% at disease onset up from 0.9±0.3% of randomly selected non (or pre)-diabetic NOD mice, **Figure 6**, left panel). This mitotic activity largely decreased to 1.5±0.5% following sustained exogenous insulin-induced normoglycemia (insulin-pellet). Overall, NOD mouse β-cell proliferation rates also correlated well with pre-euthanasia BG levels (**Figure 6** right panel, r = 0.78). Interestingly, while restoring normal BG concentrations in our mice with CTL-induced diabetes (EAD model) rapidly restored normal β-cell replication rates, NOD mouse β-cell proliferation displayed a delayed response to insulin treatment (**Figure 6**, right panel, n = 3 grey triangles). That is, NOD mouse β-cell proliferation rates did not decrease until normal blood concentrations were restored for 3–4 days (**Figure 6**, hatched open bar, open diamonds, n = 6). The few EAD model mice that failed treatment to normalized BG levels following exogenous insulin (pellets) and anti-CD8 antibody injections (**Figure 4C**, **6**, and data not shown), presumably because they had lost too many β-cells prior to treatment, also maintained increased β-cell proliferation. More interestingly, short term insulin replacement therapy in diabetic NOD mice reversed hyperglycemia, yet regularly failed to promptly normalize β-cell proliferation (**Figure 6, pellet, early**). To restore lower β-cell proliferation rates to diabetic NOD mice required several days of insulin-induced euglycemia (**Figure 6, pellet, late**). Taken together, β-cell proliferation rates generally correlated well with recent BG concentrations as objectively quantified using flow cytometry analysis.

### Systemic BG concentration does not control proliferation of islet cells other-than-β-cells in autoimmune diabetes

Mice with recent-onset autoimmune diabetes harbor a multitude of intra-islet cells that are phenotypically and functionally different. These include abundant leukocytes, reflecting the disease's inflammatory nature, endocrine cells other than β-cells (e.g. α- and δ cells), and other non-endocrine cells, including endothelial cells. We asked what effect the chronic organ injury and the inflammatory environment might have on these cell subsets in CTL-induced EAD mice. Using incorporated BrdU as assessed by flow cytometry, we examined how these various major islet cell subsets responded to glycemia change. As shown in **Table 2**, only β-cells responded to hyperglycemia by substantially increasing replication rates, as estimated by BrdU incorporation, and then decreased that proliferation back to baseline following the return to normal BG levels. In contrast, none of the other endocrine or non-endocrine islet cell subsets exhibited such dramatic, BG concentration-dependent changes. The slightly increased non-endocrine cell proliferation rates we observed (6–10 fold over naïve controls) did not display the same glycemia-dependence. Focusing on specific cell types, α- and δ-cell replication rates remained very low and of unclear significance at diabetes onset (**Table 2**), Indeed, the apparent small proliferation rate changes were evident using BrdU-labeling only, since such changes fall below the DNA-content technique's sensitivity threshold (**Figure 1, inset**). We have generated data suggesting that pancreatic α-cell numbers decrease by more than 3-fold in mice with recent onset diabetes (data not shown), and have speculated that this α-cell loss is an adaptive process, secondary to β-cell destruction and islet regenerative activity. It is therefore possible that a slightly increased α-cell proliferation reflected recovery of the α-cell compartment, and α-cell numbers further normalized after restoration of normoglycemia through α-cell replication (**Table 2**),. Since the rate of cell death and or endocrine cell differentiation from progenitor cells is unknown, we must emphasize that proliferation rate changes do not necessarily imply an absolute increase or decrease in the total number of certain cell subsets. Taken together these data suggest that elevated BG levels, occurring when autoimmune diabetes becomes clinically manifest, specifically and selectively stimulate β-cell multiplication.

**Table 2:** Blood glucose selectively controls ß-cell proliferation in islets of diabetic mice.

	naïve	Diabetes	Diabetes rx'd	Diabetes rx'd
	(n = 7)	no treatment, n = 7	+islet Tx, n = 7^a^	+s.c.−ins. pellet, n = 4^b^
**islet cell subset**	**BrdU**			
	%	% (fold incr.)	% (fold incr.)	% (fold incr.)
**insulin^+^ β cells**	0.1±0.0^c^	4.6±0.9 (55 d)	0.7±0.3 (7)	1.7±0.3 (19)
**glucagon^+^ α cells**	0.4±0.1	1.7±0.5 (3)	4.2±1.1 (10)	4.1±2.0 (10)
**somatostatin^+^ δ cells**	2.4±0.7	0.9±0.3 (0.2)	0.5±0.3 (0.2)	1.0±0.3 (0.4)
**non-endocr. islet cells** e	0.8±0.2	7.2±1.0 (8)	6.1±0.6 (6)	5.5±0.6 (6)
**CD45+ leucocytes**	n/a^f^	17.5±2.3 (n/a)	12.8±1.3 (n/a)	17.5±2.7 (n/a)

a, b average random blood glucose during BrdU labeling was (**a**) 7.6 , and (**b**) 7.5 mmol/l, respectively (see **Supporting Information Table S1**).

c BrdU+ cells among specified subset ±S.E.M.

d fold increase of BrdU+ cells over levels of naïve mice

e non-endocrine cells defined as cells that were CD45-negative and expressed neither insulin, glucagon, or somatostatin.

f naïve Rip-CD80^+^GP^+^ did not contain leukocytic islet infiltrates

## Discussion

Unlike naïve islets, islets from autoimmune diabetic animals are composed of much fewer β-cells (and perhaps α-cells, data not shown) surrounded by vast numbers of leukocytes, some of which are vigorously proliferating. The typical mouse islet structure is lost once diabetes is manifest, and these changes complicate histological analysis. Such challenges include difficulties distinguishing β-cells bound by effector CTLs, or recently killed, apoptotic β-cell debris found in nearby phagocytes [51]. Moreover, islets within the diseased pancreas display a rather heterogeneous pattern of lymphocytic infiltration. Flow cytometry overcomes many of these limitations; and in addition is quantitative, objective in nature, provides high-throughput capability, and offers the possibility of simultaneously testing multiple parameters. Employing multicolor flow cytometry approaches to dissociated islet cells, we were able to examine the proliferative properties of the individual endocrine cell subsets during development of autoimmune diabetes with a precision not previously achievable. Importantly, we found variable proliferation in the different islet cell subsets in response to immune-mediated islet destruction and diabetes development, pointing to distinct growth regulatory circuits operating on the different islet cell subsets. We found that only islet β-cell proliferation was tightly correlated with BG levels.

Numerous *in vitro* and *in vivo* models have correlated glycemia with β-cell mass [16], [29], [30], [52]–[54]. While β-cells' proliferative potential has important therapeutic implications for T1DM [55], only few experimental studies have probed that proliferative potential and the factors regulating it during β-cell autoimmunity [11], [25], [26], [56]. Reasoning from immunohistology obtained during the pre-diabetic phase in NOD mice, investigators have proposed that residual β-cell proliferation is controlled by T-mediated islet inflammation, because β-cell turnover was found to be already elevated before glycemia control was lost [25], [26]. In variance with these studies, using flow cytometry to test two independent parameters of cell proliferation, namely total DNA content per cell and BrdU incorporation, we find using two different diabetes models (CTL-induced EAD and spontaneous NOD) that mouse β-cell proliferation is sharply up-regulated coincident with overt hyperglycemia at disease onset; but much less so in the difficult to define pre-diabetic disease stages. Our interpretation that elevated BG levels at disease onset stimulate β-cell proliferation is further supported by our data showing restored normoglycemia (often despite unaltered autoimmunity) rapidly lowered β-cell replication rates to near-normal. We can suggest at least three not-mutually-exclusive explanations for the slightly higher than normal β-cell proliferation rates observed in mice with restored glycemia control after diabetes onset. One, we cannot control exogenous insulin dosing with the fine specificity required to achieve BG control close to that achieved by a healthy pancreas; postprandial hyperglycemia is particularly difficult if not impossible to control in a mouse. Thus, residual elevated β-cell proliferation rates may simply reflect pathological glucose tolerance in these mice. Our islet transplantation results support this idea since previously diabetic mice that received whole islet grafts reduced β-cell proliferation virtually to control levels in 5 of 7 mice (**Figure 4B**), and did better than subcutaneous pellet-released exogenous insulin. Two, islet β-cell loss may result not only in insulin deficiency but also in the loss of other factors important in fine tuning carbohydrate metabolism, including amylin peptide [57], glucagon-like peptide 1 or its receptor agonists (reviewed in [58]), islet-neogenesis-associated-protein [59], activin [60], [61], and betacellulin [61], and therefore exogenous insulin may not, on its own, restore all functions lost when β-cell mass falls below a critical threshold. Our islet transplantation data support this idea as well. Three, factors like the local inflammatory environment, organ injury, and/or net β-cell deficiency may have a metabolism-independent and direct effect promoting regenerative β-cell proliferation in the once-diabetic mice. The latter mechanism was proposed by Sherry et al. [26] who used immunofluorescence microscopy and image analysis to study NOD mouse β-cells, and they reported only about a 2-fold increase in ß-cell proliferation in the mid to late prediabetic phase, but before elevated BG levels became evident. These authors suggested a prominent role played by autoaggressive T cells mediating β-cell proliferation in advance of diabetes onset since they observed reduced β-cell turnover (2–3 fold) up to 6 weeks after Treg or anti-CD3 mAb therapies had down-regulated aggressive autoimmunity and restored glycemia control. An alternative explanation might be that the minor increase in β-cell proliferation we have noticed during the pre-diabetic phase (Figure 1B, and data not shown) resulted from early subclinical glucose intolerance.

While in our study both the CTL-induced EAD and the spontaneous NOD mouse models demonstrated sharply increased β-cell proliferation rates at diabetes onset, we did note a few differences between the models. Most notably, NOD mice responded to exogenous insulin induced normal glycemia by down-regulating their β-cell proliferation, but with delayed kinetics compared to the mice with CTL-induced EAD. β-cell proliferation in diabetic NOD mice (based on BrdU uptake) did not cease until a few days after glycemia control was restored (**Figure 6**). It is of interest that at disease onset, NOD mice have very few large islets containing virtually all residual β-cells and these islets are located near the pancreas core ([62] and our unpublished data), while most islet remnants show either α-, δ cells or only inflammatory cells. It is possible that these giant islets, not seen in our EAD mice, reflect the slow progression of islet autoimmunity in NOD mice, and could account for the delayed adjustment of β-cell proliferation after restoration of normal BG levels. It is further possible that the survival of residual ß-cells results from a more favorable balance in individual islets between effector lymphocytes and immune regulatory mechanisms, which locally allows β-cells to survive and sustain proliferation, and over time can grow to large islet structures. In this environment, abrupt (therapeutic) changes in BG levels perhaps do not translate into an immediate halt of β-cell turnover. In fact, continuous exogenous insulin (pellets) more frequently caused hypoglycemia in diabetic NOD in the first few days after treatment, and rarely in EAD mice (data not shown). Hypoglycemia may reflect an additive effect in the NOD mouse of exogenous insulin and temporarily continuing β-cell replication in a glucose-resistant fashion.

In conclusion, we propose a scenario in which hyperglycemia at clinical disease manifestation drives an increase in β-cell mass, mediated to a large extent by residual β-cell proliferation. Even though such newly generated β-cells would be subject to immune rejection, our data could imply a temporary net gain in β-cells at disease onset which might explain or contribute to the reduced insulin requirements patients typically experience shortly after T1DM diagnosis, commonly known as “honeymoon” [13]. The honeymoon's transience may result from the exhaustion of β-cell regenerative capacity in the presence of an unchecked anti-β-cell autoimmune response. Such a view would emphasize the potential of spontaneous recovery should the immune response be permanently halted at this clinically prominent stage.

## Supporting Information

Methods S1 (0.03 MB DOC)

Table S1 (0.04 MB DOC)

Figure S1 (0.02 MB DOC)

Figure S2 (0.18 MB DOC)

Figure S3 (0.23 MB DOC)

Figure S4 (0.10 MB DOC)

Figure S5 (0.35 MB DOC)

Figure S6 (0.54 MB DOC)

Figure S7 (0.03 MB DOC)
