# Supplementary material for: Blood Glucose Levels Regulate Pancreatic β-Cell Proliferation during Experimentally-Induced and Spontaneous Autoimmune Diabetes in Mice
Source: PLoS One. 2009 Mar 16;4(3):e4827. doi: 10.1371/journal.pone.0004827 (PMC2654100; doi:10.1371/journal.pone.0004827)
Supplement: Methods S1 — (0.03 MB DOC) [file pone.0004827.s001.doc]

**Supporting Information Methods S1**

**Immunofluorescence and Immunohistochemistry.** Pancreata were fixed overnight in Formaldehyde/Zinc fixative (Electron Microscopy Sciences, Hatfield, PA), and paraffin embedded using standard procedures. Consecutive sections (6 µm, unstained) were prepared. Sections for immunostaining were deparaffinized, rehydrated, and washed, then antigen-heat retrieved (Target Retrieval Solution, Low pH, DAKO, Carpinteria, CA). Slides were stained using a polyclonal Ab to insulin (1:300, guinea pig, DAKO), and a mouse anti-BrdU mAb (clone PRB-1 conjugated to Alexa Fluor 488, Invitrogen, Carlsbad, CA). Briefly, after blocking with 2% BSA/1% donkey serum and M.O.M. blocking reagent (Vector, Burlingame, CA), primary Abs were reacted overnight at 4ºC, followed after three washes (5 min, PBS 0.1% BSA, 0.1% Tween 20) by secondary Ab detection for 1 h using anti-guinea pig-Cy3 (Jackson ImmunoResearch) polyclonal Ab, and counterstained with DAPI for 5 min. Fluorescence analysis was performed on a Zeiss LSM 510 Confocal microscope using Zeiss LSM 510 Meta software (Carl Zeiss Microimaging, Thornwood, NY).
