# Supplementary material for: Blood Glucose Levels Regulate Pancreatic β-Cell Proliferation during Experimentally-Induced and Spontaneous Autoimmune Diabetes in Mice
Source: PLoS One. 2009 Mar 16;4(3):e4827. doi: 10.1371/journal.pone.0004827 (PMC2654100; doi:10.1371/journal.pone.0004827)
Supplement: Table S1 — (0.04 MB DOC) [file pone.0004827.s002.doc]

**Supporting Information Table S1**: Blood glucose concentrations prior to islet isolation and analysis of proliferation by flow cytometry.

|  | **blood glucose** | **concentration ^a^** |
| --- | --- | --- |
| **diabetic mouse** | EAD | NOD |
| **treatment** | CTL-induced | spontaneous |
|  | mM±SD (n) | mM±SD (n) |
| naive (non-diabetic) | 8.3 ± 0.8 (7) | 9.0 ± 2.9 **^b^** (3) |
| untreated | 20.0 ± 1.4 (8) | 18.3 ± 1.4 (8) |
| anti-CD8 mAb (responder) | 9.0 ± 0.8 (6) | nt |
| anti-CD8 mAb (non-responder) | 18.4 ± 2.5 (3) | nt |
| insulin-pellet **^c^** (responder) | 7.5 ± 0.8 (4) | 6.6 ± 0.9 (3) |
| insulin-pellet (non-responder) | none | 16.8 ± 1.7 (3) |
| insulin-pellet **^d^** (responder - BrdU delayed) | nt | 5.7 ± 1.9 (6) |
| insulin-pellet **^e^** (after pellet removal) | 22.4 ± 2.0 (3) | nt |
| islet-Tx | 7.6 ± 2.2**^C^** (7) | nt |

**nt** not tested

**none** none observed

**a** average random BG concentration during 3-day BrdU labeling.

**b** random pre-/non-diabetic NOD mice

**c** 3-day BrdU-labelling started immediately after insulin-induced
 normoglycemia.

**d-** 3-day BrdU-labelling was delayed 3-4 days after insulin-induced
 noromoglycemia.

**e** after removal of insulin-pellet
