# Supplementary material for: Blood Glucose Levels Regulate Pancreatic β-Cell Proliferation during Experimentally-Induced and Spontaneous Autoimmune Diabetes in Mice
Source: PLoS One. 2009 Mar 16;4(3):e4827. doi: 10.1371/journal.pone.0004827 (PMC2654100; doi:10.1371/journal.pone.0004827)
Supplement: Figure S1 — (0.02 MB DOC) [file pone.0004827.s003.doc]

**Supporting Information - Figure S1**

Diabetes incidence of EAD and NOD mice plotted as a function of mouse age. EAD mice (closed symbols) were induced to develop diabetes by adoptive transfer of in vitro activated GP-specific TCR-Tg CD8+ T cells (CTL), at a median age of 10.4 weeks (arrow). Both CTL-induced EAD and NOD mice were daily checked for diabetes development. Diabetes was confirmed in urine glucose positive animals by random blood glucose of > 13 mM on two consecutive days.
