# Supplementary material for: Blood Glucose Levels Regulate Pancreatic β-Cell Proliferation during Experimentally-Induced and Spontaneous Autoimmune Diabetes in Mice
Source: PLoS One. 2009 Mar 16;4(3):e4827. doi: 10.1371/journal.pone.0004827 (PMC2654100; doi:10.1371/journal.pone.0004827)
Supplement: Figure S2 — (0.18 MB DOC) [file pone.0004827.s004.doc]

**Supporting Information - Figure S2**

Calculation of islet cell subset-specific single-cell BrdU incorporation. The illustrated example shows islet cells from a diabetic EAD mouse. The following gating strategy has been applied in consecutive order for all tested mice: cell size versus granularity (top left); CD45+ cell exclusion (top center). Note that we regularly identified a small subset of cells (events) that double-stained for insulin and the hematopoetic lineage cell surface marker CD45 (usually < 5% of all insulin+ cells, top-center, upper right quadrant). These cells were considered either CD45+ cells that had phagocytosed apoptotic β cells, or represented very stable immune cell:β-cell conjugates (e.g. CTL binding to target β cells during cell-mediated cytolysis), and therefore were excluded from further analysis. Next, endocrine cell subset-specific doublets were excluded by pulse-width gating (top - right). Endocrine islet cell subsets were identified (bottom - left) and individually plotted against BrdU-staining (bottom - right). Percentages indicate the relative frequency among individual endocrine subsets. Using this approach we identified between 37 and 283 BrdU+insulin+ cells per pancreas in any of the healthy or diabetic mice tested.
