# Supplementary material for: Blood Glucose Levels Regulate Pancreatic β-Cell Proliferation during Experimentally-Induced and Spontaneous Autoimmune Diabetes in Mice
Source: PLoS One. 2009 Mar 16;4(3):e4827. doi: 10.1371/journal.pone.0004827 (PMC2654100; doi:10.1371/journal.pone.0004827)
Supplement: Figure S3 — (0.23 MB DOC) [file pone.0004827.s005.doc]

**Supporting Information - Figure S3**

Gating strategy to visualize and calculate islet subset-specific, single cell DNA content. Pancreatic islets from a representative, newly diabetic EAD mouse were isolated, dissociated, and subjected to multicolor flow cytometry. The following electronic gating procedures were consecutively applied as follows: Cell size vs. granularity (forward vs. sideward light scatter, top - left), exclusion of CD45+ leucocytes (not shown); identification of endocrine cells by insulin- (blue) and glucagon-stained cells (red, top - right); doublet exclusion using nuclear dye fluorescence distribution characteristics (Vybrant DyeCycle Violet, Invitrogen, s. Methods). Events with high overall “area” but small maximal fluorescence “peak” were considered doublets and excluded (bottom - left). Total DNA content of single cells is shown on a linear scale (bottom - right). Cells with DNA staining intensity (DNA content) greater than 1.4 x median linear fluorescence intensity of the G_0_/G_1_ peak (containing a normal 2n chromosomal DNA, large left peak) were identified and the relative frequency (percentage) calculated.
