# Supplementary material for: Blood Glucose Levels Regulate Pancreatic β-Cell Proliferation during Experimentally-Induced and Spontaneous Autoimmune Diabetes in Mice
Source: PLoS One. 2009 Mar 16;4(3):e4827. doi: 10.1371/journal.pone.0004827 (PMC2654100; doi:10.1371/journal.pone.0004827)
Supplement: Figure S4 — (0.10 MB DOC) [file pone.0004827.s006.doc]

**Supporting Information - Figure S4**

Detection of proliferation-associated nuclear antigens in β-cells by flow cytometry. β-cell were defined by gating on dispersed islet cells from naïve (left column) or diabetic mice (middle column) that stained for insulin. Increased DNA-content (x-axis) of proliferating cells during S- and G_2_/M-Phases was determined by a quantitative nuclear DNA-staining. Co-stainings are shown using either AlexaFluor 488-conjugated monoclonal mouse anti Ki-67 (clone B56, BD Biosciences, middle panel), or the M-Phase-specific, rabbit-anti-p-histone H3-AlexaFluor 488 (Cell Signaling Technology, lower panel). Ki-67 antigen could be detected in diabetic β-cells, but was not detectable in β-cells from naïve mice. In contrast, p-histone H3 could not be detected in any β-cell tested (below detection threshold). Gating the same diabetic islet cell sample on CD45+ islet infiltrating cells was used to validate antibody-staining patterns for Ki-67 and p-histone H3. Note that the latter stained a small fraction (~50-fold less than Ki-67) of cells with the highest DNA-content, containing all cells caught in the M-Phase of the cell cycle (right column, lower plot). One of two independent experiments with similar results for each staining is shown, and additional staining of a mouse β-cell tumor line (Min6) had been performed with similar results (data not shown).
