# Supplementary material for: Blood Glucose Levels Regulate Pancreatic β-Cell Proliferation during Experimentally-Induced and Spontaneous Autoimmune Diabetes in Mice
Source: PLoS One. 2009 Mar 16;4(3):e4827. doi: 10.1371/journal.pone.0004827 (PMC2654100; doi:10.1371/journal.pone.0004827)
Supplement: Figure S5 — (0.35 MB DOC) [file pone.0004827.s007.doc]

**Supporting Information - Figure S5**

BrdU incorporation in diabetic EAD and NOD mice. Newly diabetic CTL-induced EAD (left) and NOD mice (right) were pulse labeled using a single i.p. dose of BrdU ( 1.5 mg/mouse). Pancreata were harvested the following day, and examined by confocal microscopy (s. **Supporting Information Methods S1**). A few BrdU-stained nuclei of insulin+ islet cells were regularly detected in both mice (green arrow). Bar: 100 µm.
