# Supplementary material for: Blood Glucose Levels Regulate Pancreatic β-Cell Proliferation during Experimentally-Induced and Spontaneous Autoimmune Diabetes in Mice
Source: PLoS One. 2009 Mar 16;4(3):e4827. doi: 10.1371/journal.pone.0004827 (PMC2654100; doi:10.1371/journal.pone.0004827)
Supplement: Figure S6 — (0.54 MB DOC) [file pone.0004827.s008.doc]

**Supporting Information - Figure S6**

Co-localized BrdU-stained ß cell nuclei can be observed in regenerating pancreata after BrdU-labeling for 3 consecutive days. Since anti CD8 mAb treatment halts/impairs CTL-mediated ß cell killing, we tested if gradual restoration of normoglycemia following anti-CD8 mAb treatment over a periods of several days (Figure 4A) involved ß cell replication. Newly diabetic mice were anti-CD8 mAb treated, and BrdU-pulse-labeling started the following day for 3 consecutive days during this transition phase (random blood glucose during BrdU-labeling was 10.2 mM). These experiments differ from those shown in Figures 4 and 5, where BrdU-labeling was carried out several days later after stable normoglycemia was achieved (average blood glucose: 8.4 mM). Pancreata were analyzed by confocal microscopy (**Supporting Information – Methods S1**). Images A-D shows a selection of (small) islets found to contain two or more neighboring clusters of proliferating ß cells (Insulin+BrdU+, green arrows). The localization patterns of these replicating Insulin+BrdU+ cells suggest that ß cells not only incorporate BrdU but also divide at least once during the 3 day BrdU-labeling period. Note that after anti-CD8 mAb treatment, small islets appeared to contain substantially less lymphocytic infiltrate. It is possible that the (transient) elimination ß cell-specific CTLs in the presence of elevated blood glucose allow ß cells to replicate efficiently. Bar: 50 µm.
