# Supplementary material for: Blood Glucose Levels Regulate Pancreatic β-Cell Proliferation during Experimentally-Induced and Spontaneous Autoimmune Diabetes in Mice
Source: PLoS One. 2009 Mar 16;4(3):e4827. doi: 10.1371/journal.pone.0004827 (PMC2654100; doi:10.1371/journal.pone.0004827)
Supplement: Figure S7 — (0.03 MB DOC) [file pone.0004827.s009.doc]

**Supporting Information - Figure S7**

Average crossectional nuclear area of BrdU-labeled and BrdU-negative β-cell nuclei are comparable. Recently diabetic EAD mice were BrdU-labeled by single i.p. injections per day for 3 consecutive days and pancreata were harvested the following day. 6µm sections from 3 similar mice were examined by staining for insulin, BrdU, and counterstaining by DAPI. 20 randomly found insulin^+^BrdU^+^ nuclei (grey symbols) were measured each with additional 2-4 insulin^+^BrdU^-^ nuclei in the immediate vicinity of the same islet (open symbols). Lines indicate averages: BrdU^-^, 32.9±1.0 µm^2^, n=64; BrdU^+^, 32.2±1.5 µm^2^, n=20. These data does not support the notion that β-cell endoreduplication, characterized by increased DNA-content and nuclear volume, occurs with some regularity in pancreata of diabetic mice.
